# Cardiac Tamponade Risk Associated With Anticoagulation for Atrial Fibrillation in Dialysis-Associated Pericarditis: A Case Report

**DOI:** 10.7759/cureus.39072

**Published:** 2023-05-16

**Authors:** Mohammad Abu-Abaa, Mohamed Hassan, Aliaa Mousa, Hassaan Arshad, Samir Shah

**Affiliations:** 1 Internal Medicine, Capital Health Regional Medical Center, Trenton, USA; 2 Cardiology, Capital Health Regional Medical Center, Trenton, USA

**Keywords:** hemorrhagic pericarditis, hemopericardium, atrial fibrillation, pericarditis, heparin

## Abstract

Heparin is a preferred initial anticoagulant in patients with new-onset atrial fibrillation (AF). Despite continuous debate about the risk, there has been a concern about heparin-induced hemorrhagic pericarditis and cardiac tamponade. We present a case of a new onset atrial fibrillation (AF) in a patient with renal impairment and evidence of pericardial effusion complicated by hemopericardium development after starting anticoagulation. Although the risk of hemorrhagic conversion of uremic pericarditis induced by heparin in ESRD patients with new onset AF was suggested in the literature, this case raises the possibility of a similar complication in dialysis-associated pericarditis. Therefore, we aim to heighten alertness regarding this potential complication of a commonly used medication in clinical practice. We also aim to review the current anticoagulation recommendations in this setting.

## Introduction

Heparin-induced hemorrhagic pericarditis/cardiac tamponade is rarely encountered and may be easily missed. Although anticoagulation is beneficial in patients with atrial fibrillation (AF) without end-stage renal disease (ESRD), data regarding both the efficacy and safety of most anticoagulants in patients with AF and ESRD is sparse. In addition, these are largely observational, and it is important to remember that patients with renal impairment have been excluded from the major clinical trials regarding this matter [1]. In this case, we are presenting a patient with ESRD with a new-onset AF who inadvertently developed hemorrhagic pericarditis/hemopericardium in the setting of dialysis-associated pericarditis.

## Case presentation

A 54-year-old male patient presented to the emergency department (ED) for midsternal chest pressure, generalized weakness, and fatigue for one week. Past medical history was significant for end-stage renal disease (ESRD) on regular hemodialysis (HD) for several years before this presentation. He was compliant with his HD sessions. He also has a history of heart failure with preserved ejection fraction. In the ED, his physical exam was unremarkable except for an irregular peripheral pulse that proved to be a new onset atrial fibrillation (AF) on electrocardiography (EKG). No hemodynamic instability was noted. Initially, basic labs showed only mild hyperkalemia at 5.5 mEq/l. No evidence of infection was noted. EKG showed no ST changes. However, new echocardiography showed physiologically significant anterior and posterior pericardial effusion as indicated by increased respiratory variation of mitral inflow. No evidence of right ventricular (RV) or right atrial (RA) collapse was noted. It also showed a 55-60% preserved ejection fraction with grade II diastolic dysfunction.

**Figure 1:**
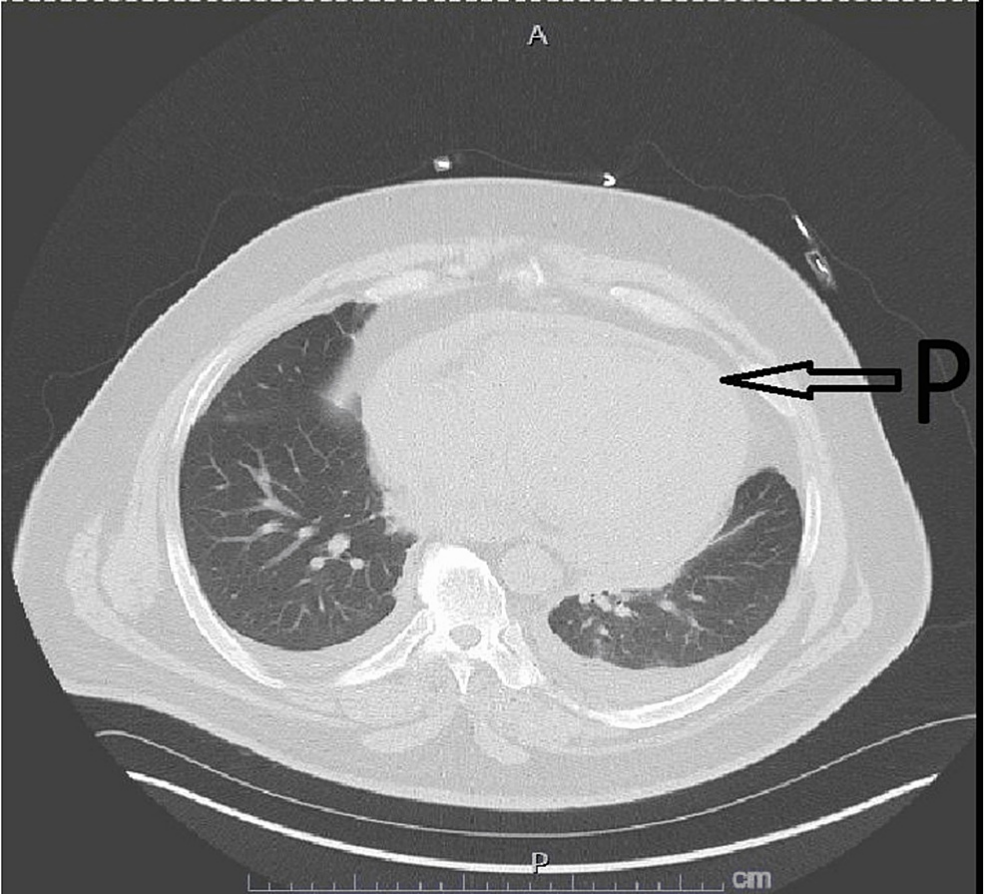
Computed Tomography (CT) Scan of the Chest Initial computed tomography (CT) of the chest upon initial presentation showed pericardial effusion (arrow).

**Figure 2:**
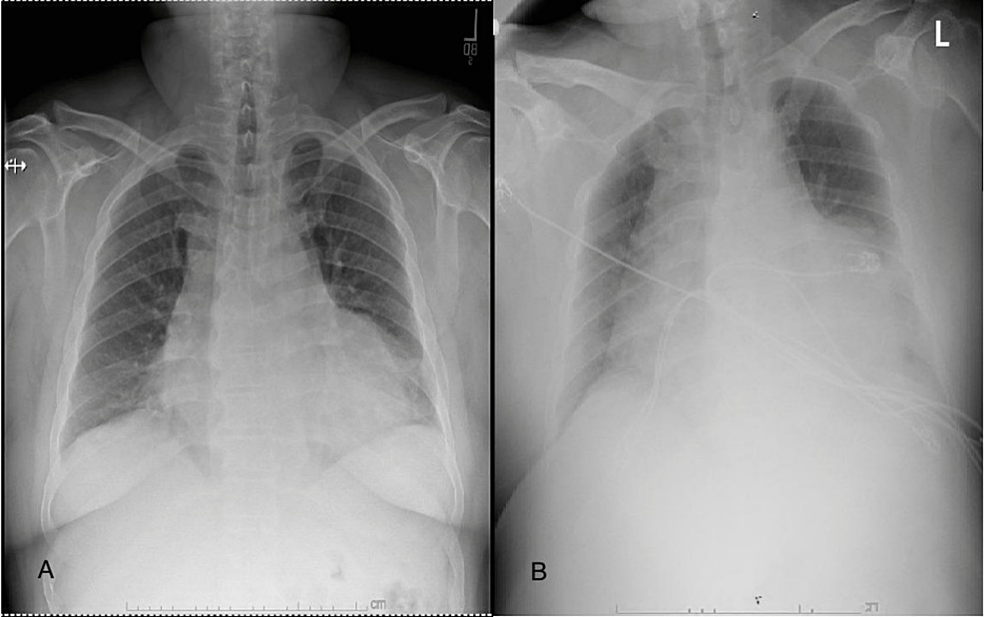
Chest X-Ray Initial chest x-ray (A) as compared to chest x-ray obtained at the onset of hemodynamic instability (B) showing increased cardiac silhouette.

**Figure 3:**
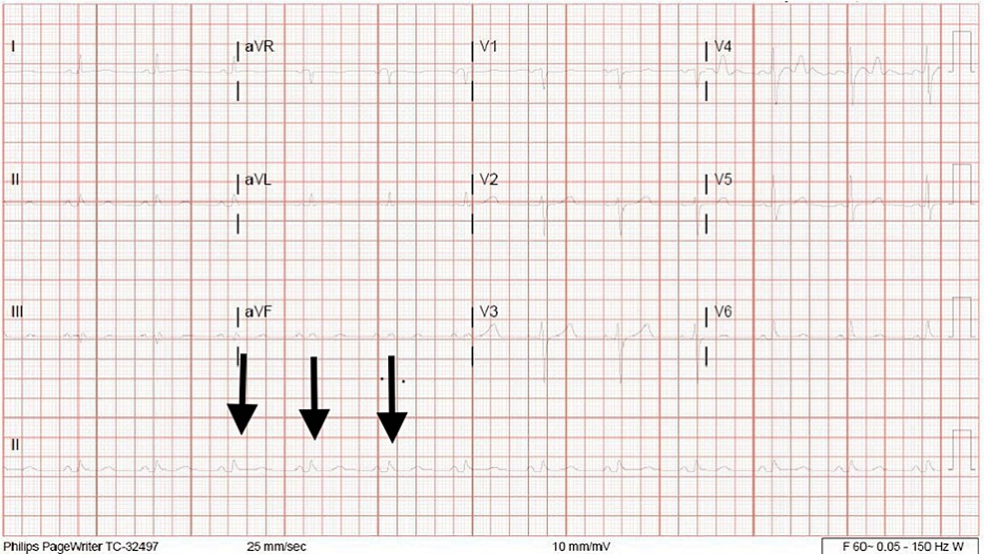
Electrocardiography (EKG) EKG showing low voltage QRS complexes in limb leads (arrows in lead 2 rhythm strip).

**Figure 4:**
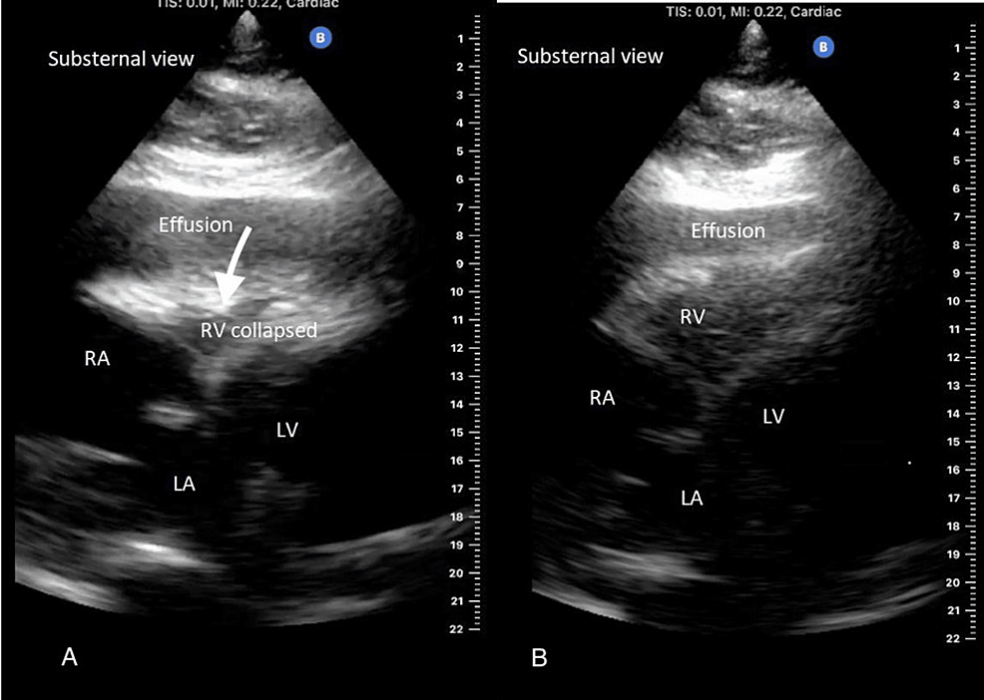
Bedside Echocardiography Bedside echocardiography using  Butterfly iQ+ - Handheld Ultrasound showing collapsing right ventricle during diastole (A) as compared to distended right ventricle during systole in (B). Abbreviations: RV- right ventricle, RA- right atrium, LV, left ventricle, LA- left atrium.

Heart rate was controlled with metoprolol 75 mg twice daily, and anticoagulation was achieved by heparin infusion. Unfractionated heparin was preferred to direct-acting oral anticoagulants (DOACs) as the need for surgical intervention was potentially anticipated. Aspirin was resumed without clopidogrel. Conservative management was recommended initially through resolving the effusion with daily hemodialysis sessions. Hemodialysis was started on the day of admission. Computed tomography (CT) scan of the chest on the second day of admission showed no significant change in the size of the pericardial effusion (Figure 1). However, on the third day of hospitalization and after his third hemodialysis session, he became hemodynamically unstable with a drop in blood pressure to 70/40 mmHg. A physical exam showed muffled heart sounds, and a bedside chest X-ray showed a significant increase in the size of the cardiac silhouette (Figure 2). JVP assessment was difficult secondary to the patient's obese habitus. EKG showed sinus rhythm but low voltage (Figure 3). Heparin was discontinued. A Stat focused cardiac ultrasound showed RV diastolic collapse (Figure 4). Activated Рartial Тhromboplastin Тime (aPTT) was only moderately elevated when the tamponade effect occurred. Two readings immediately before and after the clinical recognition of tamponade were 50-75 seconds. He was immediately brought to the operation room, and an emergent pericardial window was established.

The patient successfully underwent evacuation of 800cc hemorrhagic effusion with the improvement of vital signs. However, the recurrence of atrial fibrillation postoperatively resulted in hemodynamic instability that required intubation and vasopressor support. Further echocardiography showed resolution of pericardial effusion. The patient was successfully extubated.

## Discussion

Heparin-induced massive pericardial effusion is a rare entity that can easily be missed, and the diagnosis is not usually established until cardiac tamponade becomes clinically apparent. However, no clear incidence is documented in the literature, and a causal relationship has been debated as studies still show conflicting evidence. One series involving a cohort of 274 patients with acute pericarditis found no increased risk of hemorrhagic pericarditis and cardiac tamponade development with anticoagulation by heparin or other anticoagulants [2]. In contrast, another study involving a cohort of 453 patients with acute pericarditis indicated that patients with cardiac tamponade were three times as likely to have been on oral anticoagulants as those with no complications. Another study involving 822 patients with acute pericarditis showed no increased risk of tamponade with heparin or other oral anticoagulants [3].

Renal failure has been proven to increase the risk of patients using heparin to develop bleeding [4]. The most likely explanation of bleeding tendency in renal failure patients is a primary hemostasis defect secondary to impairment of platelet function [5]. Pericarditis in end-stage renal disease (ESRD) patients is likely to increase such risk and has been described as two entities, either uremic or dialysis pericarditis. Uremic pericarditis has been traditionally described as the onset of clinical signs and symptoms of pericarditis before initiating renal replacement therapy (RRT) or within eight weeks of its initiation. Dialysis associated-pericarditis has been defined as the occurrence of clinical manifestations of pericarditis after being stabilized on RRT for more than eight weeks. The latter type has been attributed to electrolyte changes, especially potassium level disturbance [6]. 

In the setting of renal failure-associated pericarditis, the risk of arrhythmia, including atrial fibrillation (AF), is increased [7]. In addition, the risk of embolic events is doubled among patients with renal failure who develop AF regardless of their CHA2DS2 Vasc score [7]. There has been a suggestion of an increased risk of progression of pericarditis to hemorrhagic pericarditis/cardiac tamponade if these patients are anticoagulated. No specific recommendation is suggested by the American guidelines regarding anticoagulation in pericarditis-induced atrial fibrillation in those with renal failure. On the other hand, the 2015 European guidelines recommend avoiding anticoagulation in patients with uremic or iatrogenic pericarditis who develop AF but indicate that anticoagulation may be considered in other patients with acute pericarditis-AF [8]. In addition, it is outlined in the same recommendations that the mere presence of pericardial effusion does not necessarily establish the diagnosis of pericarditis in patients with renal failure, and the diagnosis of pericarditis should be based on the clinical picture and electrocardiography (EKG) findings. In our case, and despite the lack of EKG findings suggestive of pericarditis, the suggestion of acute pericarditis was based not only on the presence of pericardial effusion but also on the presence of clinical chest discomfort at the time of presentation. As the patient had been on established hemodialysis for several years before this occurrence, this case demonstrates that heparin may still induce hemorrhagic pericarditis/cardiac tamponade in patients with dialysis associated-pericarditis. 

In our patient, In this case, new onset hypotension in the setting of heparin infusion raised the suspicion of cardiac tamponade that was further heightened by clinical findings of muffled heart sounds. Chest x-ray was a fast bedside test that confirmed the suspicion by showing an interval increase in cardiac silhouette. The most useful clinically was a bedside ultrasound. The most sensitive and specific sign of cardiac tamponade in ultrasound is right ventricle collapse during diastole, which holds 93% sensitivity and 100% specificity [9].

Heparin is preferred for anticoagulation, given the availability of an antidote in contrast to new anticoagulants. In addition to heparin, hemopericardium has also been reported in those using new oral anticoagulants (NOACs), fibrinolytic, glycoprotein inhibitors, prasugrel, and dabigatran. In this setting, idarucizumab has been useful in controlling bleeding [10]. Recently, andexanet alfa was found to be effective in reversing hemorrhagic complications of apixaban and rivaroxaban, NOACs that work through direct thrombin inhibition. It has been shown to rapidly reverse the anticoagulant effects of direct and indirect factor Xa inhibitors, such as enoxaparin and fondaparinux [11].

Regarding guidelines for anticoagulation among those with ESRD and AF with no evidence of pericarditis, due to lack of evidence, further research was called for by the European guidelines. However, the American guidelines recommend warfarin treatment for this group of patients. Recently, a meta-analysis showed that among patients with AF and mild-to-moderate renal dysfunction, subgroup analyses from trials and observational studies appear to support the net benefit of warfarin over no anticoagulation [12].

## Conclusions

Although it remains debatable recommendation to use anticoagulation in those with a new-onset AF and evidence of acute uremic pericarditis, this case raises the possibility of a similar adverse outcome of hemorrhagic pericarditis and cardiac tamponade in those with dialysis-associated pericarditis and new-onset AF when started on heparin. European guidelines recommend avoiding this approach in those with uremic pericarditis but do not offer recommendations regarding dialysis-associated pericarditis. This case points to the risk of hemorrhagic pericarditis and tamponade, even in dialysis-associated pericarditis, and if suspected, bedside ultrasound is the fastest way to confirm the suspicion.
